# Community-oriented, hospital level genetics: a new approach to improve access for underserved communities

**DOI:** 10.1038/s41390-025-03908-2

**Published:** 2025-02-19

**Authors:** Yoel Gofin, Fadel Tibi, Eliana Fanous, Shay Ben-Shachar, Rivka Sukenik-Halevy

**Affiliations:** 1 Genetics Institute, Meir Medical Center, Kfar Saba, Israel; 2 School of Medicine, Faculty of Medical and Health Sciences, Tel Aviv University, Tel Aviv, Israel; 3 Sharon Shomron District, Clalit Health Services, Tel Aviv, Israel; 4 Schneider Children’s Medical Center, Petah Tikva, Israel; 5 Clalit Innovation, Clalit Health Services, Tel Aviv, Israel

## Abstract

**Background:**

Certain populations are at increased risk for genetic syndromes but have limited access to genetic testing.

**Methods:**

We founded a community-based, pediatric genetics clinic in the Muslim-Arab city of Tayibe, Israel. Children with suspected genetic conditions of consanguineous parents, or families with two or more affected siblings were referred by local staff. The clinic was staffed by a Meir Medical Center (MMC) clinical geneticist. Blood samples were collected during the initial visit. Tests were publicly funded, with no parental involvement in administrative procedures required. A control group consisted of MMC pediatric genetics clinic patients.

**Results:**

During the first year, 30 children were assessed. No patients were lost to follow-up, compared to 8 (28%) in the MMC control group. The average time to test results was shorter in the Tayibe group and the diagnostic rate was higher, with 27.6% receiving a diagnosis (42.9%, excluding autism cases).

**Conclusion:**

Our first-year experience shows the success and promising results of this model, with advantages in almost all parameters, compared to the traditional, hospital-based clinic. Factors such as faster time-to-results, greater family adherence and satisfaction, and zero lost to follow-up rate suggest considering implementing this model for providing genetic services to other underserved populations.

**Impact:**

- A community-oriented approach for a pediatric genetics clinic allowed reaching high-risk populations, with increased adherence, faster results and a higher yield.
- Our clinic relied solely on available public funding and staff, requiring no additional contributions.
- The current dogma of hospital-based genetics services should be reconsidered.

## Introduction

The odyssey to obtaining a genetic diagnosis is often preceded by various challenges, not all of which are related to the likelihood of achieving a molecular diagnosis. These include difficulties related to patient adherence, mainly due to insufficient knowledge about the availability of genetic testing^1^ or not understanding the benefits of testing,^2^ as well as technical issues related to lack of access to genetic testing—either physical^3^ or financial.^4^

Israel provides universal healthcare coverage, funded by a compulsory, progressive health tax.^5^ A set of services covers various genetic tests according to clinical criteria, such as Chromosomal Microarray Analysis (CMA), Fragile X testing, next generation sequencing gene panels^6^ and periconceptional carrier screening tests. Exome sequencing is also publicly funded, according to specific criteria.

Most genetic work-up in Israel is performed within the public health system, mainly in hospital-based genetic clinics. This is also true in other countries, including Australia, France, and Norway.^7^ While video counseling is gaining popularity in Israel, the initial assessment for pediatric cases requires an in-person physical evaluation in a hospital-based genetics clinic.

In Israel, the Arab minority is about 21% of the population.^8^ Tayibe is the fourth largest Arab city in Israel, with a population of over 46,000,^9^ consisting almost entirely of Muslim-Arabs. The city’s socioeconomic status is in the 3rd decile, on a scale of 1–10 (where 1 is the lowest and 10 is the highest).^10^ While the rate of consanguinity in Muslim communities in Israel has decreased over the years, it is still relatively prevalent. In a 2010 survey, 24% of couples from Tayibe who were included in the survey were from consanguineous families.^11^ Consanguinity is well-known to increase the risk for genetic syndromes.^12^

**Fig. 1:**
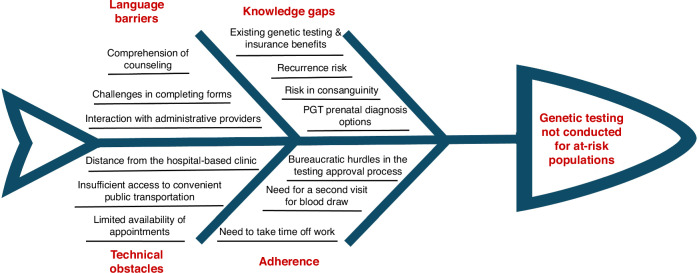
Ishikawa diagram, mapping the factors behind the gap in genetic work up usage in underserved Muslim-Arab communities, such as in Tayibe. IVF in vitro fertilization, PGT Preimplantation Genetic Testing.

Tayibe is located about 16 km from Meir Medical Center (MMC), the nearest hospital. Although close, many Tayibe-based families who would theoretically benefit from genetic testing, do not arrive for scheduled clinic visits. Given the current conditions, a significant gap exists between the availability of state-funded genetic evaluations and their utilization in underserved communities, such as Tayibe, Israel. There are several factors that play a role in this (Fig. 1). To address these issues, we established a hospital-level community genetics clinic in Tayibe as an extension of our hospital genetics clinic. Our goal was to close the existing gap by providing genetic services within the community we serve, ensuring that families can complete the genetic work-up. This includes managing all aspects of testing, including tasks typically handled by the patients’ families, such as insurance approval requests. In this paper, we share our experience with a clinic that provides community-based, accessible genetic services for an underserved population. Our aim is to illustrate a framework that can be implemented in various similar contexts, both within Israel and beyond.

## Materials and methods

**Fig. 2:**
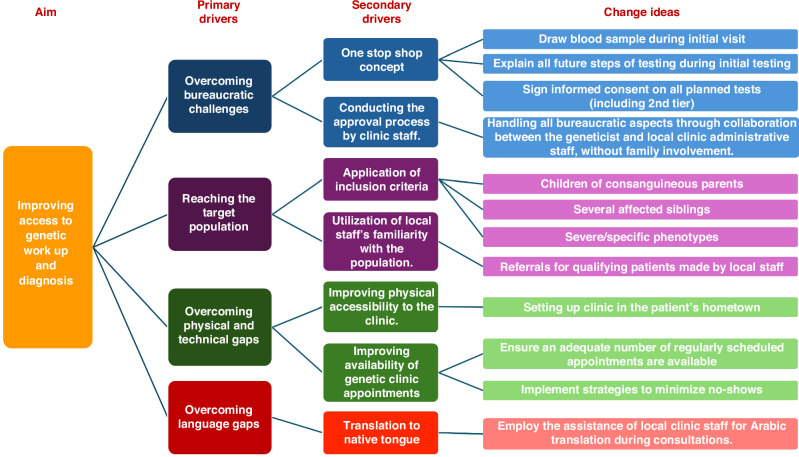
Driver diagram.

The pediatric genetics clinic in the Tayibe Center for Child Health was founded in March 2023. The Center for Child Health is the city’s community clinic for children insured by Clalit Health Services, the largest health maintenance organization in Israel.^13^ The clinic was managed and staffed by a certified geneticist from MMC (Y.G.), who works in close coordination with the local medical team on patient selection, and all clinical and administrative requirements. All cases that were seen from March 2023 through February 2024- the first year of the clinic- were included in this analysis. With the clinic’s goal of improving access to genetic evaluations and diagnoses, its various components addressed the gaps that are present in the traditional model of genetic work up and counseling (Fig. 2).

The clinic’s inclusion criteria were children under 18 years of age, with intellectual/neurocognitive impairment (including autism) and/or congenital anomalies, who were either born to consanguineous parents or had one or more siblings (under 18 years, as well) affected by the same medical condition. The Tayibe medical team referred children meeting these criteria based on their knowledge of the local population. Referrals were then prioritized according to the geneticist’s clinical assessment.

The clinic was designed according to the one-stop shopping concept, where all necessary activities take place during a single initial visit, including genetic consultation, explanation regarding the entire genetic work-up process, obtaining informed consent and blood sampling. All administrative tasks, which are usually the responsibility of the patient’s family, are performed by the clinic’s teams, including obtaining approval for testing from the health insurer and the Ministry of Health. Arabic-Hebrew translation was provided whenever needed.

A control group consisted of children who were seen at the MMC pediatric genetics clinic the week before or after the Tayibe clinic, by the same clinician (Y.G.), on the same day of the week. This group included children from a variety of ethnic origins and diagnoses.

We compared several parameters between the Tayibe clinic and the control group from the MMC hospital clinic and designed the interventions to improve these parameters within the Tayibe clinic (Table 1). Children from families where the mother was pregnant were excluded from the analysis because these are usually placed on a more urgent schedule, with a faster turnaround time.

**Table 1:** Outcome measures and the interventions designed to improve them.

Outcome measure	Tayibe clinic intervention
Number and type of genetic tests conducted	One-stop concept that encompasses blood sampling and obtaining informed consent during the initial visit, followed by the clinic staff managing the insurance approval process.
Number of patients who were lost to follow-up (did not complete recommended testing)	
Testing turnaround time (time from genetic consultation to receipt of test results)	
Number of patients who missed their scheduled appointment	Local pediatric clinic staff contact families and explains the importance of the appointment (in Arabic).
Number of patients diagnosed with a genetic condition	Inclusion criteria of consanguineous parents or multiple affected children in sibship

To assess the parents’ view of the community-based genetics service, the parents of the children from the Tayibe clinic were asked to complete a multiple-choice questionnaire written in both Arabic and Hebrew (Supplemental material S1), which included specific questions regarding the Tayibe clinic.

### Statistical analysis

Chi-squared test was used to test the differences between the use of genetic testing in the case and control groups. Two-sided, type 2 student t-test was used to measure the turnaround times for genetic testing in the two groups. A *P* value less than 0.05 was considered statistically significant. Python version 3.5.1 was used to analyze the data and to create the plots using the following libraries: numpy, scipy.stats, matplotlib and pandas.

## Results

During the clinic’s first year, 30 patients from 19 families underwent a genetic evaluation in the clinic. The control group consisted of 29 cases from MMC. Patients’ characteristics are described in Table 2.

**Table 2:** Patients’ characteristics.

Characteristic	Tayibe clinic *n* = 30	Meir medical center comparison group *n* = 29
Families, number	19	26
Consanguinity, n (%)	12 (63.2)	1 (3.8)
Sex		
Female, n (%)	14 (46.7)	10 (34.5)
Male, n (%)	16 (53.3)	19 (65.5)
M:F ratio	1.14	1.9
Average age at first presentation, years [range]	5.1 ± 4.1 [0.2–17]	6.8 ± 4.7 [1.5–17.8]
Muslim-Arab Patients, n (%)	30 (100)	6 (20.7)
Indication for genetic counseling, n (%)*		
Neurodevelopmental abnormality and/or autism	25 (83.3)	19 (65.5)
Congenital anomalies	5 (16.7)	3 (10.3)
Seizures	2 (6.7)	3 (10.3)
Microcephaly	6 (20)	2 (6.9)
Short stature	2 (6.7)	5 (17.2)
Muscle weakness	1 (3.3)	2 (6.9)
Other	1 (3.3)	2 (6.9)

*Since some patients had several indications, the percentage is greater than 100.

**Fig. 3:**
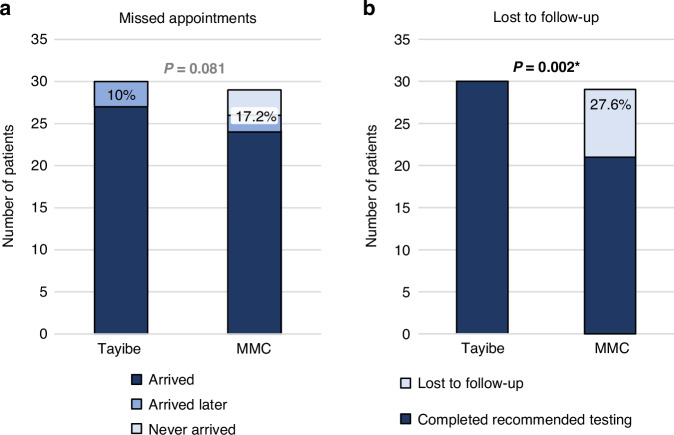
Comparison of attendance and adherence between the Tayibe clinic and the MMC control group. **a** Patients who did not arrive at their scheduled appointments. **b** Patients who did not complete recommended genetic testing. Significant *p* values are marked with an asterisk.

Three patients from two families missed their appointment in the Tayibe clinic (10% no-show rate; Fig. 3a). All three were rescheduled and later completed assessment, bringing the actual no-show rate to zero. Among the controls, the no-show rate was 5 of 29 (17.2%), with only 2 of these children later receiving genetic counseling.

All Tayibe clinic patients completed recommended genetic testing, and none were lost to follow-up. This was not the case for the control group where 28% did not complete recommended testing and were lost to follow-up (Fig. 3b).

**Fig. 4:**
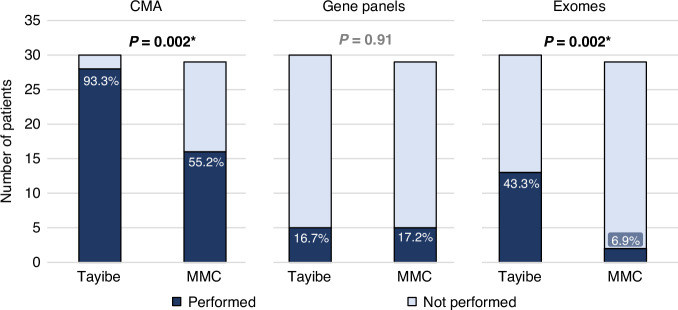
Genetic tests completed by patients. The Tayibe group included 5 patients who had a CMA prior to the clinic (prenatal) and the MMC group included 4. Significant *p* values are marked with an asterisk.

Of the 30 Tayibe clinic patients, 28 (93.3%) completed CMA testing, 5 (16.7%) had gene panel testing and 13 (43.3%) had exome sequencing. In the MMC control group, the percentage of patients who completed a CMA or an exome was significantly lower (Fig. 4).

**Fig. 5:**
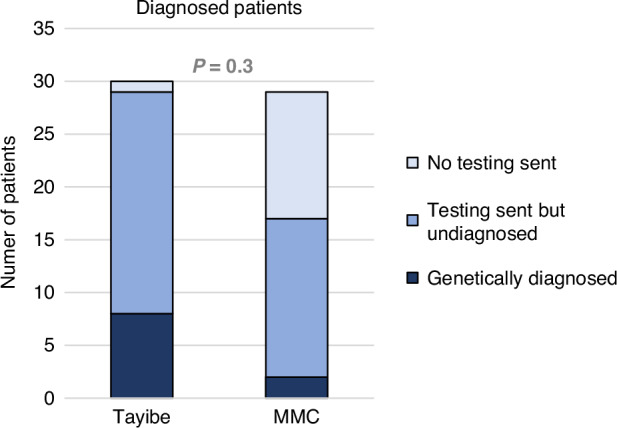
Genetic diagnosis rates.

Twenty-nine patients in the Tayibe clinic underwent genetic testing (one patient did not undergo testing due to technical difficulties). Seven patients had positive genetic testing results (Fig. 5), with 5 diagnoses (two couples of siblings carried the same variants). One additional patient had a pathogenic incidental finding (Table 3, classification of variants based on ACMG criteria^14^), bringing the diagnostic rate in the Tayibe clinic to 27.6%, more than twice the rate in the control group (11.8%, *p* = 0.3). Among the 29 Tayibe clinic patients who had genetic testing, 15 were diagnosed with autism, of whom only one had a causative genetic diagnosis, and another patient had an incidental finding. When excluding these 15 patients, the diagnosis rate was higher, 42.9%. The 8 diagnosed patients included two cases with homozygous variants in genes that cause an autosomal recessive disorder (consanguineous parents), two that had a de novo variant in an autosomal dominant disorder and two pairs of siblings had an autosomal dominant disorder, inherited from a parent.

**Table 3:** Genetic diagnoses of Tayibe clinic patients.

Syndrome	Gene/Region, inheritance pattern	Variant*	Transcript	Heterozygous/Homozygous	Inheritance	ACMG classification
Congenital myopathy 3 with rigid spine (OMIM #602771)	*SELENON*, AR	c.1315 C > T, p.Arg439*	NM_020451.3	Homozygous	U	PVS1, PM3, PM2
Diaphanospondylodysostosis (OMIM #608022)	*BMPER*, AR	c.1672 C > T, p.Arg558Ter	NM_001365308.1	Homozygous	U	PVS1, PM2, PP4
Coffin Siris Type 1 (OMIM #135900)	*ARID1B*, AD	c.6144_6148dup, p.Asp2050fs*49	NM_001374828.1	Heterozygous	DN	PVS1, PS2, PM2
White-Sutton syndrome** (OMIM #616364)	*POGZ*, AD	c.2310 C > A, p.Tyr770*	NM_015100.4	Heterozygous	P	PVS1, PM2, PP4
Exostoses, multiple, type 1*** (OMIM #133700)	*EXT1*, AD	c.2101 C > T, p.Arg701Ter	NM_000127.3	Heterozygous	DN	PVS1, PS4, PM2
1q21.1 Distal recurrent deletion syndrome** (OMIM #612474)	1q21.1, AD	chr1:146,488,131-147,830,830	N/A	Heterozygous	M	

*AR* autosomal recessive, *AD* autosomal dominant.

U- Unknown, the diagnosis was made using a gene panel. Only the patient was tested. Both parents were presumed to be carriers, testing was recommended. DN- De novo. P – Paternal. M- Maternal.

*Genome build: hg19/GRCh37.

** Diagnosed in 2 siblings.

*** Incidental finding.

**Fig. 6:**
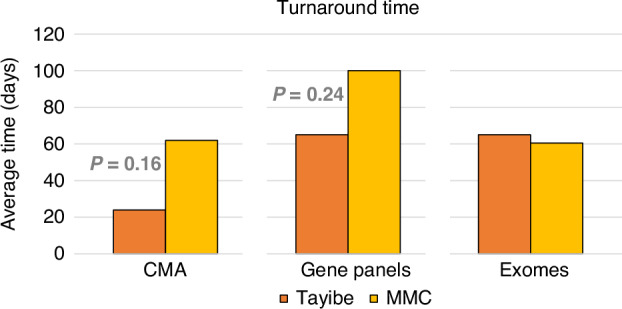
Time (in days) between the day of consult/result of previous test and the day of the test report, for CMA, gene panels and exomes. Of note, the small number of completed exomes in the control group did not allow calculation of a *P* value.

The average turnaround time (Fig. 6) for CMA and gene panels was much shorter for the Tayibe clinic compared to the hospital-based clinic (24 days vs. 62 days for CMA, *P* = 0.16 and 65 days vs. 100 days for gene panels, *P* = 0.24). The turnaround time for exome sequencing was 65 days for the Tayibe clinic compared to 60.5 days for the hospital clinic (of note, the control group contained only 2 patients who underwent exome sequencing).

All 19 families from the Tayibe clinic completed the questionnaire. Six of 17 families (35%) had intentions to expand their families. With regards to information-seeking prior to the appointment in the Tayibe clinic, 63% received information regarding the counseling from their physician, 26% from a family member and 26% searched for information online. When asked about a previous referral for genetic counseling, 58% had received a recommendation in the past and 37% had tried to schedule an appointment. When asked why genetic assessment was not completed in the past, the most prevalent reasons were lack of interest and lack of a referral by a physician (32% for each). Seventy-eight percent of respondents stated that they would have arrived at MMC for genetic counseling if they had a scheduled appointment. Families were asked to assess the amount of information given to them during the genetic consultation, to which 47% responded it was too much, and 53% said it was adequate (no family responded receiving too little information).

## Discussion

This paper describes our experience obtained during the first year of a novel, hospital level community-oriented Pediatric Genetics Clinic in the Muslim-Arab city of Tayibe, Israel. While the concept of providing genetic services to underserved populations in order to overcome disparities in the genetics field is not new, in similar initiatives, patients were seen in a large medical center^15^ or via telemedicine-based clinics, as in the Texome project in Texas, USA.^16^ Also, these programs are usually funded by various organizations, such as the National Human Genome Research Institute or from philanthropic donations.^15,16^ The novelty of the Tayibe clinic is that it is linked to a hospital-based genetics institute; thus, relying on existing public funding and personnel. It does not require any additional resources and is physically located in the community it serves. In addition, the model on which the clinic was built was focused on removing major technical obstacles and simplifying the process of genetic work-up. The concept of moving the testing location to a clinic near the families’ homes (even where the physical distance from the hospital clinic is minimal) and providing this service in a familiar location with familiar staff who can help deliver accessible information in their native language is, in our view, an important change that resulted in the success of this clinic.

The Tayibe clinic had better results compared to the MMC clinic (which represents the current traditional method of providing genetic services in Israel) on almost every parameter examined. More tests were completed in the Tayibe clinic with faster turnaround times and with no patient lost to follow-up during the evaluation process. The ability of the clinic staff to take care of all administrative aspects allowed the families to focus on the genetic counseling. In addition, the fact that the parents were not required to secure prior approval for testing or to deliver reimbursement forms, allowed us to overcome a significant barrier that had caused some patients in the MMC clinic to abandon genetic work-up, especially in populations with language barriers and lower socioeconomic status. Of note, the control group included families of various socioeconomic backgrounds. We assume that had the MMC control group included only families with low socioeconomic status, the differences between the groups would have been larger (due to lower adherence and testing utilization in these populations^17^).

Another significant advantage to the Tayibe clinic is the one-stop shop approach. A study published in 2020, based on a five-year experience with a county genetics clinic,^18^ showed that loss to follow-up during the phlebotomy stage was the most common reason for tests to not be completed. Our comprehensive approach circumvents that risk, and we believe it is one of the main reasons that none of the patients were lost follow-up. The financial challenges mentioned in the 2020 USA study, are less relevant to our clinic, due to the benefits of Israeli public health insurance and the Ministry of Health’s exome funding, which provided coverage for all tests.

A genetic diagnosis was found in 27.6% of patients tested in the Tayibe clinic. It is not surprising that the diagnostic rate is higher for this group compared to the control group, since the Tayibe group was highly selected and we believe the higher rate reflects the clinic’s success in reaching its target population, which has higher risks for genetic syndromes. The diagnostic rate in the Tayibe clinic was even higher when looking specifically at the patient who did not have a diagnosis of autism. It seems that even in our selected population, autism was a relatively low-yield indication for Next Generation Sequencing testing. This is similar to what is known in the literature.^19^ It is worth mentioning that in Israel, exome sequencing for patients with autism who also have a sibling with that diagnosis is covered by the Ministry of Health, which was a consideration for seeing these patients in the Tayibe clinic.

The patients with the homozygous variants in *SELENON* and *BMPER* were referred to specialized clinics that provide targeted supportive care (such as respiratory), and their parents were counseled regarding the option of preimplantation genetic testing to lower their current 25% risk of recurrence (autosomal recessive disorders) in future pregnancies. Other patients who were diagnosed in the Tayibe clinic were referred for further evaluations (such as imaging and hearing tests), according to the standard recommendations related to their newly diagnosed syndromes. Importantly, genetic diagnosis provided accurate evaluations of recurrence rates in the immediate family.

By focusing on a Muslim-Arab community, we were able to bridge some existing gaps for this minority group, which has lower utilization rates of specialist care.^20^ By selecting cases with a high likelihood for a monogenic genetic condition, we can reach a population of patients with a higher yield from genetic testing. The fact that children were referred by the local medical team, and that the clinic is in the patients’ hometown, allowed to overcome some of the barriers noted in our survey, including the low motivation for genetic counseling and the difficulty to physically attend clinics in the hospital.

The platform presented in this paper may be suitable for many other patient groups that have similar difficulties that inhibit them from achieving genetic counseling and testing. We suggest that this model can be easily reproduced and implemented in additional locations. This may be even more significant when this type of clinic is planned for populations with higher diagnostic yield, such as consanguineous families. Also, the technical obstacles that families experience in securing funding approval for testing are very limiting. Hence, transforming the existing hospital-based clinics into one-stop-shop clinics, even without changing their location, may lead to a substantial improvement in adherence and decrease lost-to-follow-up rates.

As this report describes the clinic’s first year, the numbers presented are still too small to show statistical significance in most of the parameters measured. However, we believe the existing data show promising results. Although the clinic is advantageous in most parameters, some aspects are more challenging for the medical geneticist, mainly the inability to receive real-time, firsthand, clinical input from colleagues at the MMC clinic. However, the use of patient photographs and clinical group discussions may provide sufficient clinical support to the medical geneticist in charge of this type of outpatient clinic.

Our clinic model serves as a pilot that can be replicated in other scenarios throughout Israel and beyond. We believe that the success of such replication hinges on several key elements. We have found that the most crucial factor is cooperation with local clinic staff, both medical and administrative. This collaborative partnership is essential throughout the entire genetic diagnosis process, starting from patient selection and continuing through to results delivery and necessary follow-up.

Another important component is the support from the medical center, which enables this innovative model that requires greater effort and flexibility from the center’s staff. Additionally, the presence of universal medical coverage acts as a significant facilitator for the model’s effectiveness. In underinsured or uninsured populations, additional resources may be necessary for successful implementation.

After the clinic’s first year, we recognize that the sustainability of our clinic depends on the commitment of both parties - the medical center and the local clinic. We also acknowledge that showcasing our achievements within our organizations and on public platforms, such as meetings (and this publication), helps promote the project and generates interest from other institutions in adopting this model. This interest has led us to recently open a second clinic in a different location, following the same model.

To maintain sustainability, it is also crucial to actively seek new patient candidates after the initial- and likely more recognizable ones- have been seen. This can be achieved by engaging with physicians in the local community and reaching out to specialized clinics that serve patients from the targeted region, such as child development centers.

## Conclusion

This community-based quality improvement approach for genetic counseling and testing shows promising results, with advantages in almost all parameters tested. The results indicate a need to reconsider the currently existing model used to provide genetic services in many countries and healthcare systems, where counseling is performed primarily in large hospitals. This approach would increase the availability and provision of genetic testing to underserved populations, with minimal additional funding and resources. This proposed model can be replicated in various additional settings, allowing other underserved communities to benefit from it, worldwide.

## Supplementary information

Supplementary file 1

## Data Availability

The datasets generated during and/or analyzed during the current study are available from the corresponding author on reasonable request.
