# Supplementary material for: Community-oriented, hospital level genetics: a new approach to improve access for underserved communities
Source: Pediatr Res. 2025 Feb 19;98(4):1276–82. doi: 10.1038/s41390-025-03908-2 (PMC12549321; doi:10.1038/s41390-025-03908-2)
Supplement: Supplementary file 1 [file 41390_2025_3908_MOESM1_ESM.pdf]

Supplementary file 1

Parent Questionnaire

Please complete each question.

1. Male/female.
2. Age of parent.
3. Are you (the parents) related to each other?
    1. First-degree cousins.
    2. Further consanguinity.
    3. Same Surname but no known relationship.
    4. Different Surname and no known relationship.
4. How many children do you have?
5. How many of your children have the diagnosis for which you've come for a consult today?
6. Do you plan to have more children in the future? Yes/No
7. Did your child receive a genetic consult in the past? Yes/No
8. Following the referral to this clinic, did you receive an explanation from a medical professional? Yes/No
9. Following the referral to this clinic, did you receive an explanation from a non-professional person (family/friend)? Yes/No
10. Following the referral to this clinic, did you read medical material online? Yes/No
11. Did you receive a recommendation to complete a genetic evaluation for your child in the past?

    If so-

1. Did you try making an appointment? Yes/No
2. Did you receive an explanation about the benefit of such evaluation? Yes/No

12. Did you receive a genetic consult in the past? Yes/No
13. Did a family member receive a genetic consult in the past? Yes/No
14. If your child did not receive a genetic consult in the past, what is the reason (more than one option can be chosen)?
    1. Distance of clinic from home.
    2. Lack of transportation.
    3. Inability to miss workdays.
    4. Lack of interest/do not see the benefit of genetic work-up.
    5. Difficulty with Hebrew.
    6. I did not receive a recommendation for a genetic consult.
15. If you would have received an appointment for genetic consult in Meir Medical Center, would you have arrived? Yes/No
16. Do you think your child can benefit from a genetic evaluation? Yes/No
17. Do you think a genetic evaluation can prevent the birth of another child with the same medical condition your child has? Yes/No
18. Do you intend to complete the genetic tests that were recommended during today's consult? Yes/No
19. If the blood test for your child had been at Meir Medical Center, would you have completed it? Yes/No
20. If the genetic reason for your child's condition is found, would you use the information for in vitro fertilization? Yes/No

21. If the genetic reason for your child's condition is found, would you use the information for genetic testing during pregnancy, using chorionic villi sampling/amniocentesis? Yes/No
22. Are you satisfied with arriving to today's consult? Yes/No
23. What helped you understand today's consult? Rate each part from 1 to 4:
    1. Translation to Arabic.
    2. The available time for the consult.
    3. Visual aids.
    4. Explanation about genetic tests using the analogy to books and pages.
24. Did the consult include:
    1. Too much information?
    2. Too little information?
    3. Enough information?
